# Spatial spread of malaria and economic frontier expansion in the Brazilian Amazon

**DOI:** 10.1371/journal.pone.0217615

**Published:** 2019-06-18

**Authors:** Patrícia Feitosa Souza, Diego Ricardo Xavier, Martha Cecilia Suarez Mutis, Jurema Corrêa da Mota, Paulo Cesar Peiter, Vanderlei Pascoal de Matos, Mônica de Avelar Figueiredo Mafra Magalhães, Christovam Barcellos

**Affiliations:** 1 Oswaldo Cruz Foundation, Institute of Scientific and Technological Information and Communication in Health, Health Information Laboratory, GIS Laboratory, Rio de Janeiro, Brazil; 2 Oswaldo Cruz Foundation, Oswaldo Cruz Institute, Rio de Janeiro, Brazil; Instituto Rene Rachou, BRAZIL

## Abstract

The temporal and spatial evolution of malaria was described for the postfrontier phase of the Brazilian Amazon in 2003–2013. The current ecological study aimed to understand the relationship between spatial population mobility and the distribution of malaria cases. The study identified epidemiologically relevant areas using regional statistical modeling and spatial analyses that considered differential infections and types of work activities. Annual parasite incidence (API) in the region was highest in hotspots along the Amazon River and in the south and west settlement zone of Hiléia, with concentrations in environmental protection areas and açaí and Brazil nut extraction areas. The dispersal force decreased in the Central Amazon due to rapid urbanization and improved socioeconomic conditions for workers in consolidated settlement areas. The study characterized the spatial patterns of disease transmission according to the economic activity and regionalization of geographic areas, confirming that the incidence of infection by work activity and labor flow is linked to extractive activities and agricultural settlements.

## Introduction

In 2015, Brazil reported the lowest number of malaria cases in 40 years. The disease has resurfaced in tropical regions in recent decades due to accelerated population growth and expanded occupation of areas bordering forests [1]. Even with occasional outbreaks in other regions of the country, the vast majority of malaria cases in Brazil (99.8%) are concentrated in the Amazon basin, which contains the country’s largest remnants of equatorial forests, where several factors favor the transmission of diseases and hinder their control.

The predominant incidence in the Amazon basin results from a series of regional socioenvironmental characteristics and the process of a region’s occupation. The natural environment is more favorable for the proliferation of anophelines (the vector mosquito species for malaria) due to the high year-round heat and humidity and dense forests and bodies of water covering much of the territory [2] [3]. Malaria is thus one of the main causes of morbidity in the Brazilian Amazon, although incidence rates vary between municipalities.

The Legal Amazon has experienced intense and uncontrolled exploitation of natural resources. It is an environmentally heterogeneous area with diverse land use, such as industrial mining, agroforestry (organized settlements and various sizes of farms and ranches), extractive reserves, and home to riverine and indigenous peoples [4], where important malaria outbreaks occur. These economic activities have resulted in intense, continuous deforestation and urbanization and increased internal migration from other regions of Brazil, facilitated by transportation networks connected to remote, previously unconnected areas [5] [6] [7].

The Brazilian Amazon is an endemic region for malaria. It consists of 807 municipalities in the states of Acre (AC), Amapá (AP), Amazonas (AM), Maranhão (MA), Mato Grosso (MT), Pará (PA), Rondônia (RO), Roraima (RR), and Tocantins (TO). In 2010, the region’s total population was 25,469,352 inhabitants. Among the 807 municipalities in these states, 52 border seven countries: Bolivia, Colombia, Guyana, French Guiana, Peru, Suriname, and Venezuela.

The concept of border or frontier malaria was coined to describe how processes of territorial occupation of the Brazilian Amazon contributed to expanded incidence of the disease [8]. According to the author, the spread of border malaria involved three stages of territorial occupation. The first stage was marked by a rapid increase in malaria incidence in the early years of occupation caused by deforestation, agricultural settlement projects, construction of local roads, substandard housing conditions, and the migrant population’s lack of knowledge concerning the disease. The second stage showed a progressive decline in malaria rates due to improved environmental and socioeconomic conditions in agricultural settlements three years after developing land occupation fronts. Finally, the third stage was one of relative stability, with lower malaria rates resulting from urbanization, an increased sense of community in the population, improved socioeconomic conditions, and a reduction in environmental changes.

Population mobility is an important global issue for the circulation of certain diseases, especially the dynamics of vector-borne diseases due to both vector circulation and entry of susceptible populations into transmission areas (in addition to carrying the parasite over long distances) [9]. These areas require spatial and temporal studies and analyses to elucidate the distribution of malaria cases.

The study of the determinants of malaria incidence in economic frontier expansion areas requires approaches aimed at assessing both the occupational conditions and the spatial context in which people become infected [10] [11] [12]. Malaria in the Amazon has been studied to identify social [13], environmental, and climatic factors [14], while research on the impact of economic macrodeterminants of malaria is rare [15]. The current study thus aimed to analyze the contextual characteristics that describe the main economic processes in territorial occupation in relation to the spatial and temporal distribution of malaria in the Brazilian Amazon from 2003 to 2013.

## Materials and methods

### Study area

In this study, the Brazilian Amazon frontier refers to regions where human settlement is still occurring, marked by the construction of communications networks, emergence and growth of towns and cities, human migration, and shaping of a new landscape [16]. Parts of the Amazon are still affected by these activities, with different stages of government policy implementation and private investment, producing extensive heterogeneity and regional disparities.

**Fig 1:**
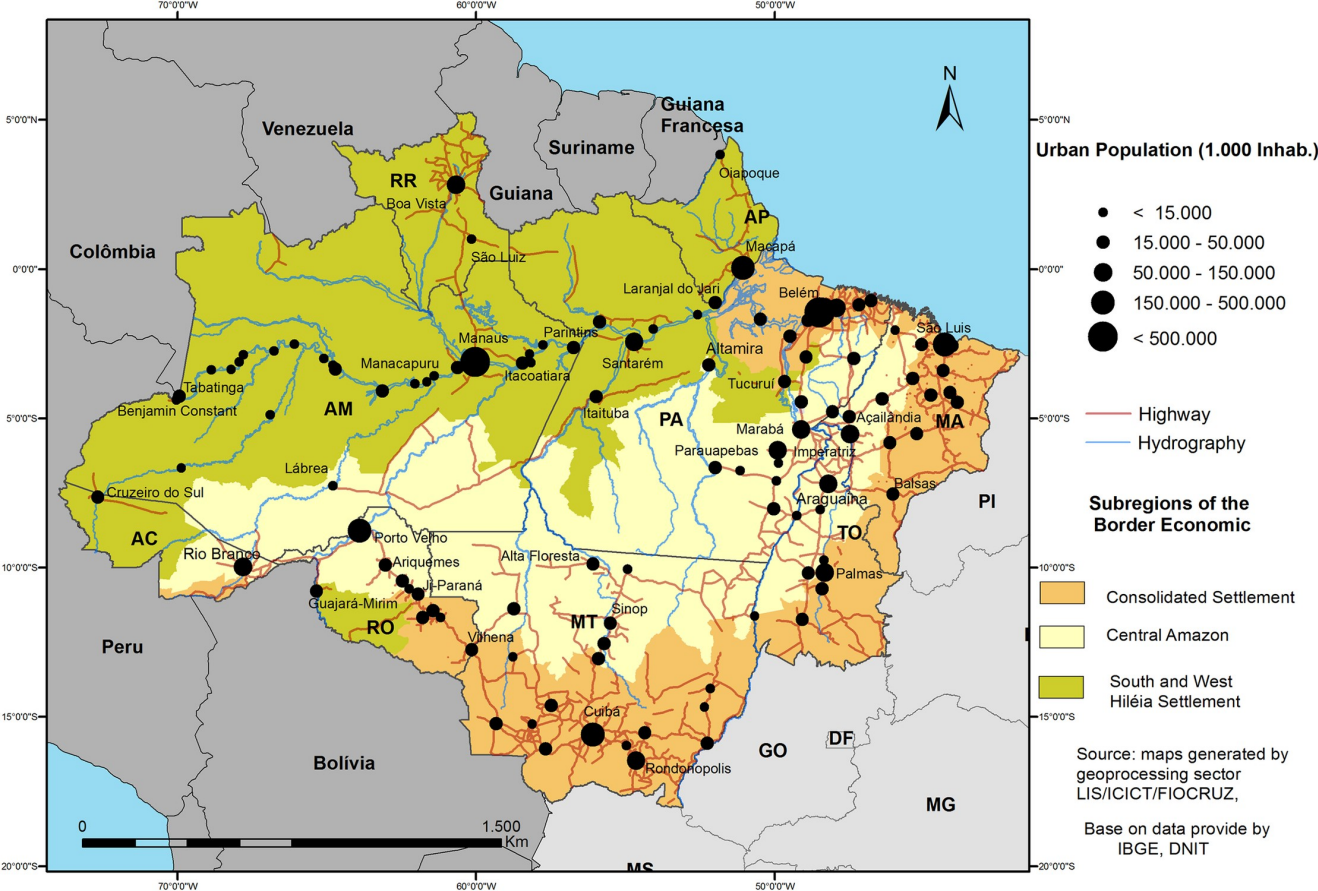
Range of economic frontier occupation: cities, highways, and waterways.

In an attempt to regionalize the economic frontier in different zones of territorial occupation, the region was divided accordingly [17] to study the relevant temporal and spatial dynamics in the expansion of the economic frontier and to contextualize and discuss the relationship between the spatial distribution of economic activities and the spatial dispersion of malaria in the postfrontier phase. This was especially important because the economic frontier is subject to dynamic changes and can shift or dissolve in the territory. The reconfiguration and transformation of territorial organization in the Brazilian Amazon are now in the postfrontier stage. The postfrontier should also be seen as a recontextualization of the Amazonian geographic space, as new areas are incorporated with contemporary technologies, practices, and institutions that regulate, characterize, and restructure the frontiers [17]. The frontier consolidation sequence is defined [18] as a phase of neoliberal land occupation in postfrontier areas with a high percentage of business ownership and strong international links to agribusinesses. Thus, we divided the range of economic frontier occupation into three major subregions: the consolidated settlement zone, the Central Amazon, and the south and west Hiléia settlement zone (Fig 1).

The consolidated settlement zone includes six areas: Belém, southeastern and eastern Pará, Tocantins, Mato Grosso, and Rondônia, all located along the southeastern and northeastern portions of the region. Much of the subregion’s economy is based on logging, cattle ranching, and soybean production. The subregion is highly dynamic, with a dense urban and roadway network consisting of medium-sized cities and state capitals [19].

The Central Amazon includes some municipalities in the states of Acre, Amazonas, Maranhão, Pará, Tocantins, and Rondônia and is located between the Cerrado (savannah) and Amazon biomes. The subregional economy specializes in logging, livestock, soybean production, and mining. It has the second highest population density of the studied areas (more than 16 inhabitants per km^2^) and a high urbanization rate [19].

The south and west Hiléia settlement zone includes some of the municipalities of the states of Acre, Amapá, Amazonas, Pará, Roraima, and Rondônia and is located in the northern and northwestern portions of the Amazonian Hiléia. The economic structure of the subregion is concentrated on forestry (açaí and Brazil nuts), logging (timber and firewood), fishing, and subsistence agriculture (manioc, bananas, and livestock, mainly water buffalo and beef cattle). The subregion’s urban network is sparsely populated, consisting mainly of small towns and a low population density (less than 1 inhabitant/km^2^). It contains the Amazon River valley, which is still an important axis for human settlement [19] [20].

### Theoretical and conceptual model

**Fig 2:**
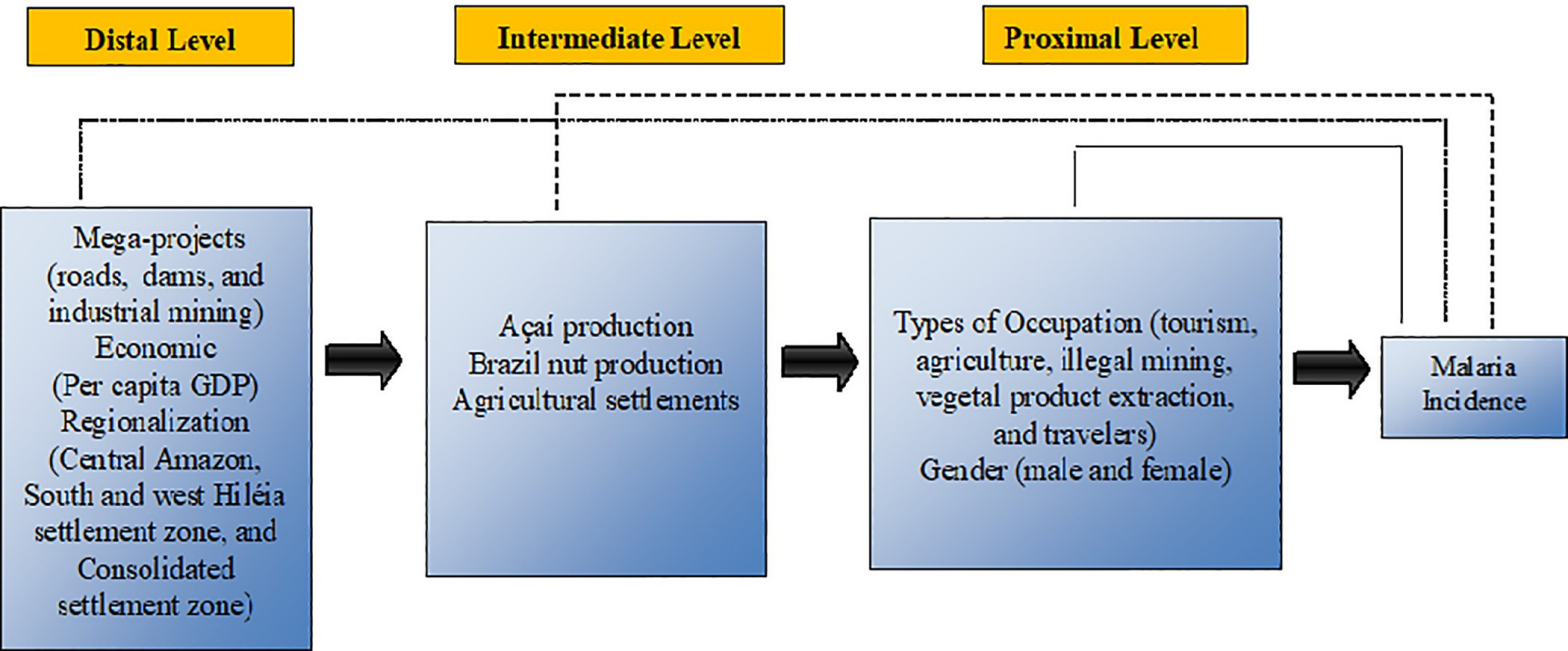
Theoretical and conceptual ecological model for the relationship between socioenvironmental determinants and malaria in the Brazilian Amazon.

A three-tier theoretical and conceptual model (Fig 2) was developed to study associations between the socioenvironmental determinants of malaria at the distal, intermediate, and proximal levels. The conceptual model is based on the social determinants of health (SDH) approach. In light of existing knowledge, the distal level included economic drivers embodie as large projects (highways, dams, hydroelectric power plants, tourism, mining, hunt and fisheries, and large-scale agriculture), per capita GDP and territorial occupation areas [21] [22] [9] [13] [23] [24]. The intermediate level included malaria exposure proxies such as the extraction of açaí and Brazil nuts as well as family farm settlements [25] [26] [24]. The proximal level included individual characteristics that influence malaria risks, such as the total number of men and women with malaria, and types of occupation (agriculture, tourism, mining, vegetal extraction and traveling) [22] [3] [14] [27] [23].

### Data collection

Malaria data were obtained from the Malaria Epidemiological Surveillance Information System (SIVEP-Malaria) and retrieved by the Health Surveillance Department of the Brazilian Ministry of Health (SVS/MS). The absolute number of cases diagnosed per year (2003–2013) in each of the 771 municipalities of the Brazilian Amazon were considered, and the municipality where the infection occurred was used as the territorial reference.

Potential factors associated with the occurrence of malaria were extracted from several sources and calculated according to municipality:

- per capita gross domestic product (GDP): mean aggregate value per capita of goods and services produced in a geographic space during the target year, measured in local currency and at market prices and based on data from the Brazilian Institute of Geography and Statistics (IBGE) [20] [28];
- Number of malaria-positive individuals related to agriculture, tourism, gold mining, vegetal product extraction, other, mining activities, traveling, and road and dam construction, obtained from the Ministry of Health (SIVEP);
- Number of men and women with positive test results for each species of *Plasmodium* analyzed and total malaria rates, obtained from the Ministry of Health (SIVEP);
- açaí production: the total amount (tons) produced from *açaí* extraction, obtained from the Automatic Data Retrieval System (SIDRA) of the Brazilian Institute of Geography and Statistics (IBGE) [20] [28];
- Brazil nut production: the total amount (tons) of Brazil nuts produced from Brazil nut extraction, obtained from the SIDRA system of the Brazilian Institute of Geography and Statistics (IBGE) [20] [28];
- human settlement: total number of settled families, provided by the regional reports by states of the Brazilian Amazon in the General Information System on Agricultural Settlements of the National Institute of Colonization and Agrarian Reform [29];
- regionalization: consolidated settlement zone in the Central Amazon (arc of deforestation) and the south and west Hiléia settlement zones.

The study was approved in June 2015 by the Institutional Review Board of the Oswaldo Cruz Foundation (CAAE 44507015.8.0000.5241), case review 1.109.048.

### Spatial mapping

The municipal digital grids for the years 2005 and 2013 were used to construct the maps. The municipal grids and the population data with geographical coordinates of the municipal seats were retrieved from the IBGE website.

Health and sociodemographic indicators were georeferenced in the municipal seats and organized in layers in a Geographic Information System environment for the construction of thematic maps.

The annual parasite incidence (API) was interpolated for spatial diffusion maps. API spatial distribution estimates were calculated using the inverse distance weighting (IDW) [30] [31] [32] geostatistical method, an application of the ArcGIS version 10 software (Esri, Redlands, CA, USA), which estimates the value of an indicator in nonsampled areas based on samples from neighboring municipal seats.

A map representing the space-time trend of malaria transmission in the Amazon region was created by interpolating the value of the epidemic year for each municipality. In this study, an epidemic onset was considered the first year in the series from 2003 to 2013 in which the number of cases exceeded the value of 10.

### Statistical analysis

A spatial-temporal study on the occurrence of malaria was developed using four specific years of SIVEP data: 2003, 2005, 2010 and 2013. These years were selected to analyze the factors associated with the incidence of malaria in the year preceding an epidemic (2003), in an epidemic year (2005), in the year in which the Millennium Development Goals were defined by the federal government (2010) and in the year after the implementation of the goals (2013). The latter year was used to assess the possible impact of the MDGs on malaria prevention and control.

Since the majority of the malaria cases were caused by *Plasmodium Falciparum* and *Plasmodium vivax*, we opted to analyze the determinants of each of these two species as well as the determinants of malaria as a whole. This strategy was used because municipalities simultaneously presented overlapping *Plasmodium* species. Therefore, the socioenvironmental determinants of the different species were analyzed. The data were disaggregated and then recombined to monitor and analyze the overlapping malaria cases.

Three dependent variables were constructed: 1) total number of individuals who tested positive for *P*. *falciparum*; 2) total number of individuals who tested positive for *P*. *vivax*; and 3) total number of malaria cases (regardless of *Plasmodium* species).

The construction of study-dependent variables was based on the *Plasmodium* species and the various combinations reported in SIVEP. The system differentiates individuals who have been infected by a single protozoan specie or infected by more than one specie, namely, *P*. *falciparum*, *P*. *falciparum* +*P*. *falciparum* gametocytes, *P*. *vivax*, *P*. *falciparum + P*. *vivax*, *P*. *vivax + P*. *falciparum* gametocytes, *P*. *falciparum* gametocytes, *Plasmodium malariae*, *and P*. *falciparum + P*. *malariae*.

The analysis was conducted by municipality, which, in Brazil, constitutes a regional political-administrative division and, in the case of the present study, contained SIVEP records for one or more *Plasmodium* species, i.e., the same municipality could have more than one species of malaria. Previously, an analysis was performed to verify which were the most frequently reported *Plasmodium* species in the system to construct the dependent variables for the study. The distribution of data showed that in Brazil, *P*. *vivax* and *P*. *falciparum* accounted for 80.0% of the total malaria reports in SIVEP. Under this scenario, we opted to analyze which of the selected determinants were related to each of the two most common *Plasmodium* species in Brazil, as well as analyze how these determinants are associated with the different species in the same locality.

Three dependent variables were constructed: 1) the sum of individuals who tested positive for *P*. *falciparum*; 2) the sum of individuals whose tested positive for *P*. *vivax*; and 3) the total number of cases of malaria (regardless of *Plasmodium* species). In this last dependent variable, malaria case totals included *P*. *falciparum*, *P*. *falciparum + P*. *falciparum* gametocytes, *P*. *vivax*, *P*. *falciparum + P*. *vivax*, *P*. *vivax + falciparum* gametocytes, *P*. *falciparum* gametocytes, *P*. *malariae*, and *P*. *falciparum + P*. *malariae*.

An initial descriptive approach using bivariate analysis was followed by an analytical method using multivariate analysis with zero-inflated binomial regression.

In the descriptive approach, the proportion of municipalities with positive malaria results for, positive results for *P*. *falciparum* and positive results for *P*. *vivax* were calculated by year. In the bivariate analysis, the means with the respective standard deviations of malaria cases by year and type of regionalization were calculated.

The total number of *P*. *falciparum* and *P*. *vivax* cases according to regionalization of the municipalities was organized in a historical series from 2003 to 2013 as well as by the contextual variables of economic production (açaí extraction, Brazil nuts, and agricultural settlements).

Since the dependent variables (number of *P*. *falciparum*, *P*. *vivax*, and total malaria cases) were set as nonnegative integer count data with no upper limit, with a large numbers of zeros and large dispersion of values, we decided to apply negative binomial regression models. This model was chosen because it provided an alternative to excessive zero-count data where there is usually data overdispersion [33] [27].

On the basis of the endemic levels of malaria in the resident population of the municipalities of the Legal Amazon, a control diagram was constructed in the following stages: 1) identification of the number of years with malaria cases exceeding 10; 2) identification of the year with the highest occurrence of cases; 3) calculation of the annual average incidences as well as their respective standard deviations; 4) calculation of the endemic upper limit, considering the added mean plus two times the standard deviation; and 5) identification of an endemic outbreak defined by the statistical significance of the Poisson test using the maximum number of cases in the period and the endemic upper limit.

### Regional analysis

Concerning the association between contextual variables (per capita gross domestic product (GDP), açaí production, Brazil nut production, and human settlements), the analysis was divided according to the theoretical conceptual model (Fig 1), namely, at the distal, intermediate, and proximal levels, with hierarchical modeling. Before modeling, a nonparametric correlation analysis was conducted to assess potential multicollinearity between independent variables. Contextual variables with correlations greater than 0.60 were excluded from the multivariate analysis, as in the case of hunting and fishing activities.

In the modeling strategy, all variables were introduced directly at the distal level, and only those with p values less than 0.05 were maintained in the model. A similar procedure was used for the intermediate level. For inclusion at the proximal level, only the variables from the distal and/or intermediate levels that were significantly associated with the outcome (p < 0.05) were maintained. This process was applied according to *Plasmodium* species and total malaria cases by region and year.

## Results

In 2003, 292,590 malaria cases were confirmed, of which 53,057 (18.1%) were *P*. *falciparum* and 226,478 (77.1%) were *P*. *vivax*. There were a total of 419,095 confirmed malaria cases in 2005, of which 87,931 (21%) were *P*. *falciparum* and 311,317 (74.3%) were *P*. *vivax*. There were 236,243 confirmed cases in 2010, of which 30,293 (12.8%) were *P*. *falciparum* and 198,587 (75.4%) were *P*. *vivax*. The year 2013 had a total of 164,484 cases, of which 20,882 (12.7%) were *P*. *falciparum* and 134,032 (81.5%) were *P*. *vivax* (Table 1).

**Table 1:** Number of municipalities with Malaria cases records by *Plasmodium* species between 2003 and 2013.

*Plasmodium* species	Year	Municipalities with confirmed cases	Total confirmed cases
***P*. *falciparum***	2003	369 (47.9%)	53,057
	2005	349 (45.3%)	87,931
	2010	263 (34.1%)	30,293
	2013	161 (20.9%)	20,882
***P*. *vivax***	2003	542 (70.3%)	226,478
	2005	506 (65.6%)	311,317
	2010	435 (56.4%)	198,587
	2013	346 (44.9%)	134,032
***Plasmodium* (all species)**	2003	560 (72.6%)	292,590
	2005	524 (68.0%)	419,095
	2010	449 (58.2%)	236,243
	2013	390 (50.6%)	164,484

Source: Malaria Epidemiological Surveillance Information System (SIVEP-Malaria)/Health Surveillance Department, Brazilian Ministry of Health (SVS/MS) [23]

The malaria results suggest there was a decrease in the number of municipalities affected by malaria as a whole. For *P*. *falciparum*, the proportion of municipalities with confirmed cases reached 47.9% in 2003 and decreased to 20.9% in 2013. For *P*. *vivax*, approximately 70% of the municipalities reported cases in 2003, decreasing to 44.9% in 2013.

**Fig 3:**
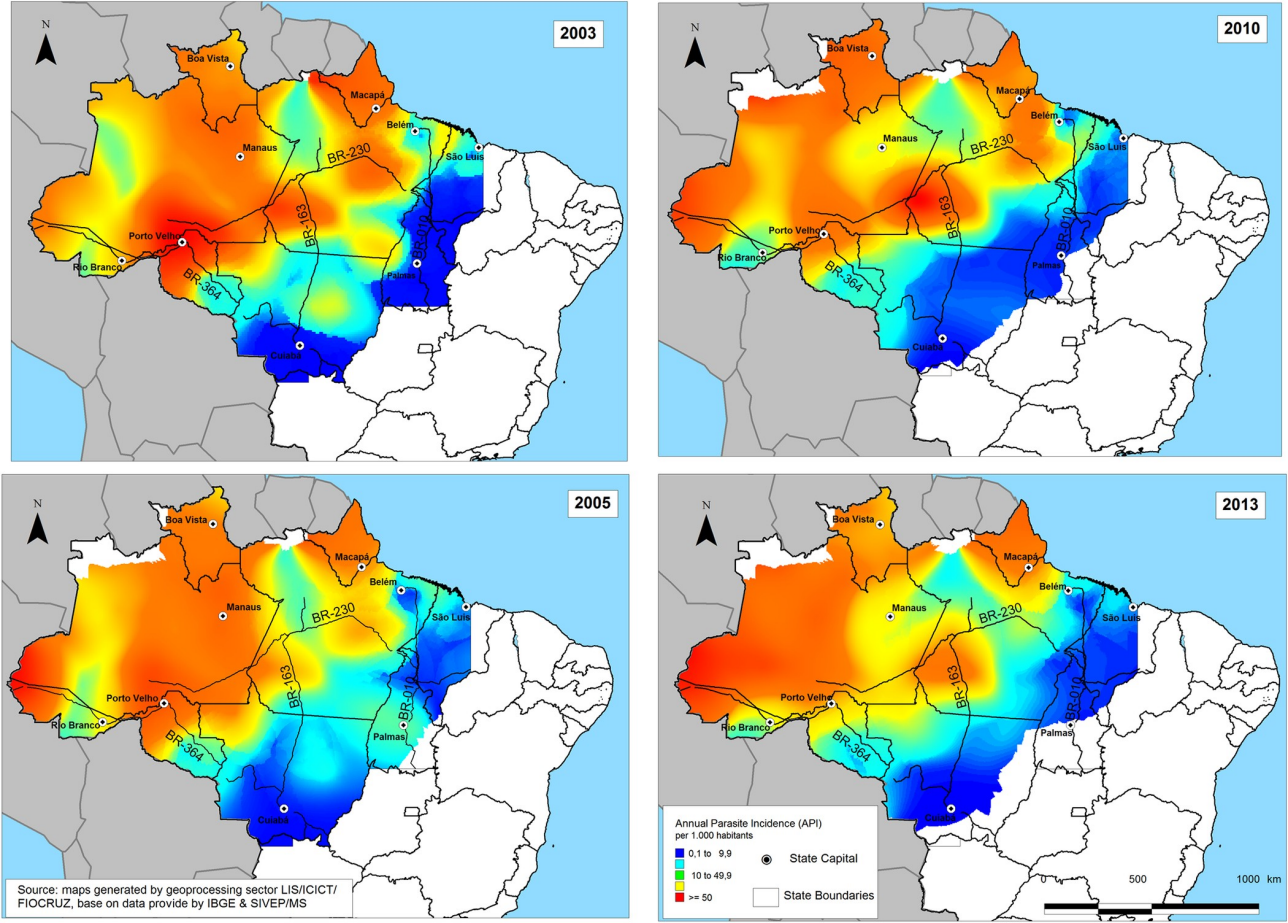
Spatial spread of annual parasite incidence (API) (2003–2013). Color codes correspond to API values per 1,000 inhabitants from 2003 to 2013.

The malaria distribution in the economic frontier was not homogeneous (Fig 3). The consolidated settlement zone had a high population density, well-functioning public services, large towns and cities, and an extensive road system. The area was not contributing to the spread of malaria in the region due to the reduction in areas serving as natural habitats for vector reproduction.

The Central Amazon had a high population density and the most economically dynamic areas in the region, specializing in export commodities. Malaria incidence was high in western Pará (northern portion of the Brazilian Amazon), northern Mato Grosso (south-central portion of the Brazilian Amazon) and Rondônia (southeast of the Brazilian Amazon). This area was the main corridor for human migration within the Brazilian Amazon to and from other areas and disseminated the disease, thus constituting a prime location for the occurrence and perpetuation of malaria epidemics.

The landscape in the south and west Hiléia settlement zone consisted mainly of forests with few urban areas and little human habitation [34]. Even with a lower population than the other zones, malaria incidence was high for all four years of the study, and the epidemic in 2005 was particularly concentrated in this region.

**Fig 4:**
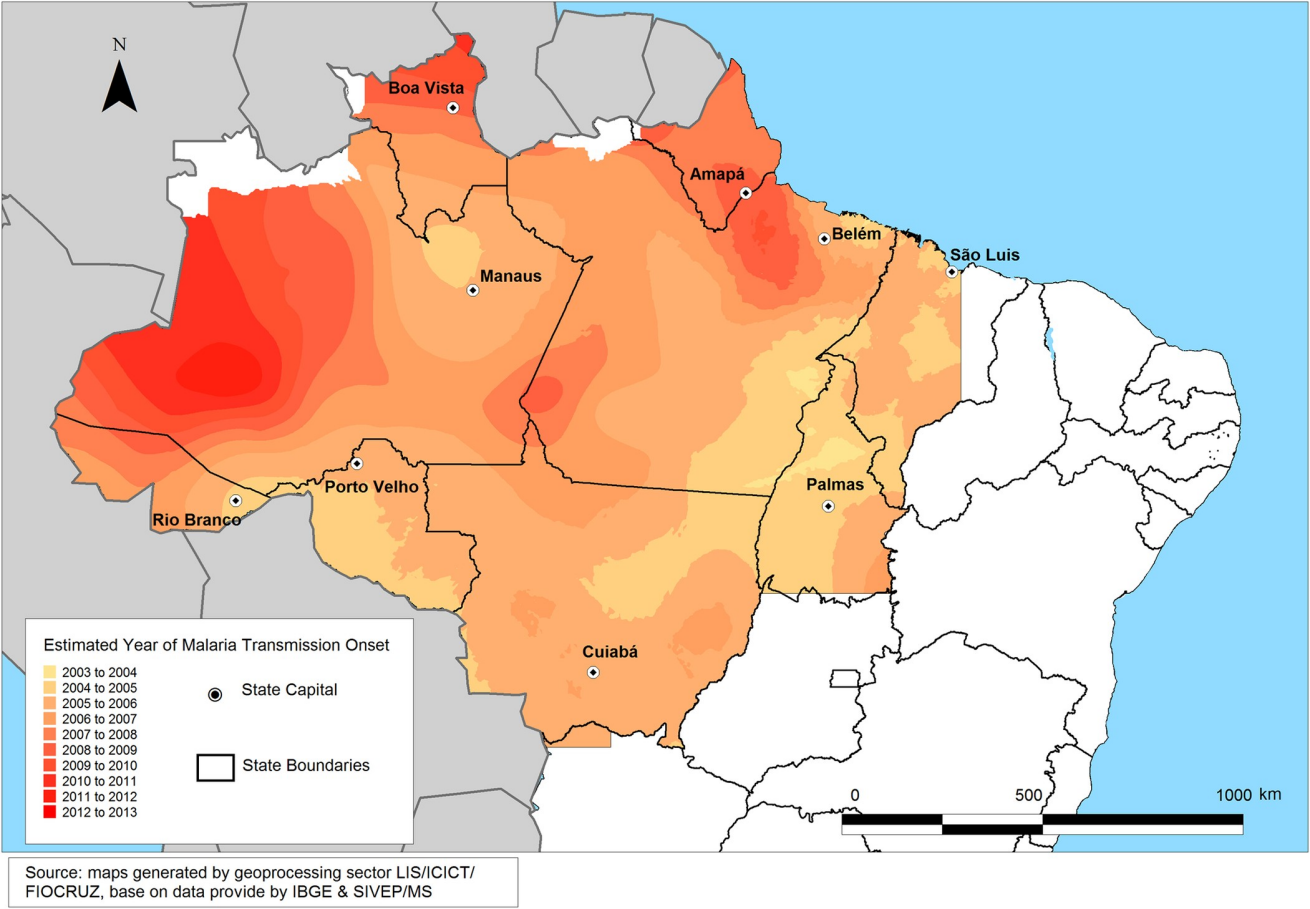
Time trend of sites with high malaria incidence in the Brazilian Amazon (2003–2013).

Fig 4 represents the evolution of the spatial distribution of malaria epidemics in the Amazon region. Lighter colors represent areas where epidemics occurred in the early years of the studied period, and darker colors represent areas where epidemics occurred in more recent years. According to the map, malaria epidemics shifted from the eastern and southern Amazon borders toward the western areas. From the map, it can be seen that pioneer fronts were responsible for malaria expansion during the beginning of the decade, while the more recent malaria cases were concentrated in remote areas with specific economic projects, environmental protection areas, vegetal products extraction areas and deforestation fronts were established.

As shown in Table 2, the effects of malaria as a whole (independently of *Plasmodium* species) on occupation types differed according to annual disease trends and type of regionalization. In the Central Amazon (the so-called arc of deforestation), the occupied areas with the highest case incidence in the epidemic year 2005 were associated with distal determinants such as agriculture (203.9 ± 896.2), industrial mining (1.5 ± 14.5), and proximal or individual level such as illegal mining (30.6 ± 285.1) Areas with tourism and road/dam construction (distal determinants) showed peak mean incidence rates in 2010 (7.3 ± 103.1 and 5.4 ± 87.8, respectively).

**Table 2:** Annual Parasite Index (API) according to regionalization, year, and occupation of the municipalities.

Region / Year	Occupation						
	Agriculture	Tourism	Illegal mining	Vegetal products extraction	Roads and dams	Industrial mining	Travelers
Central Amazon*							
2003	169.8 (671.9)	2.1 (12)	22.8 (207.9)	15.3 (87.6)	0.7 (3)	1.1 (4.4)	3.7 (19)
2005	203.9 (896.2)	4.2 (37.8)	30.6 (285.1)	11.4 (58.8)	0.8 (3.4)	1.5 (14.5)	3.5 (20.4)
2010	75.4 (377.6)	7.3 (103.1)	25.4 (374.7)	4.5 (34.4)	5.4 (87.8)	1.3 (17.2)	2.3 (12.8)
2013	21 (126.7)	2 (19.7)	20.8 (356.7)	1.3 (8.1)	1.2 (14.8)	0.2 (2.4)	1.5 (9.8)
South and west Hiléia settlement*							
2003	285.7 (856)	6.6 (39.2)	36.4 (187.1)	8.9 (22.5)	1.1 (4.5)	3.7 (9.9)	29.4 (241.2)
2005	470.9 (954)	9 (45.6)	37.1 (165)	15.2 (47.9)	3.2 (10.7)	3.1 (9.1)	57 (508.3)
2010	259.5 (529.5)	8.6 (38.7)	39.8 (236.2)	5.6 (17)	3.7 (18.4)	1.1 (3.1)	14.3 (40.2)
2013	169.2 (323.4)	4.3 (13.8)	22.3 (100.9)	3.5 (14.2)	2.4 (7.9)	0.5 (1.4)	10.3 (20.4)
Consolidated settlement*							
2003	16.8 (73.2)	0.3 (1.6)	0.2 (0.8)	8.5 (71.2)	0.3 (1.5)	0.2 (1.5)	1.5 (7.6)
2005	27 (151.9)	0.4 (2)	0.5 (3.6)	12.3 (127.3)	0.2 (1)	0.2 (1.2)	1.4 (7.6)
2010	20.8 (120.7)	0.7 (4.9)	2.6 (42.2)	11.5 (117.6)	0.1 (0.8)	1.3 (17.6)	2.1 (13.2)
2013	2.3 (12.2)	0.1 (0.5)	0.1 (0.3)	0.8 (6.9)	0 (0.2)	0 (0.1)	0.4 (2.4)

Note: The mean and standard deviation of API was considered. P-value < 0.05.

* P-value <0.05 for the student's t-test of means.

Source: Malaria Epidemiological Surveillance Information System (SIVEP-Malaria)/Health Surveillance Department, Ministry of Health (SVS/MS). [23]

There was an important decrease in API values for all of the areas and types of occupations in municipalities, especially for agriculture and the Central Amazon. The reduction was less in the south and west Hiléia settlement zone. The consolidated settlement zone showed the lowest mean API, with the exception of agriculture. In the south and west Hiléia settlement zone, in the epidemic year 2005, the activities with the highest mean malaria incidence were due to indivividual level determinants such as agriculture (470.9 ± 954), tourism (9.0 ± 45.6), vegetal product extraction (15.2 ± 47.9), and travelers (57 ± 508.3), while in 2010, the highest activities were illegal mining (39.8 ± 236.2) and road and dam construction (3.7 ± 18.4).

As for the consolidated settlement zone, the activities with the highest incidence were practically at the proximal or individual level, as agriculture (27 ± 151.9) and vegetal product extraction (12.3 ± 127.3), while in 2010 they were tourism (0.7 ± 4.9), illegal mining (2.6 ± 42.7), industrial mining (1.3 ± 17.6), and travelers (2.1 ± 13.2).

**Fig 5:**
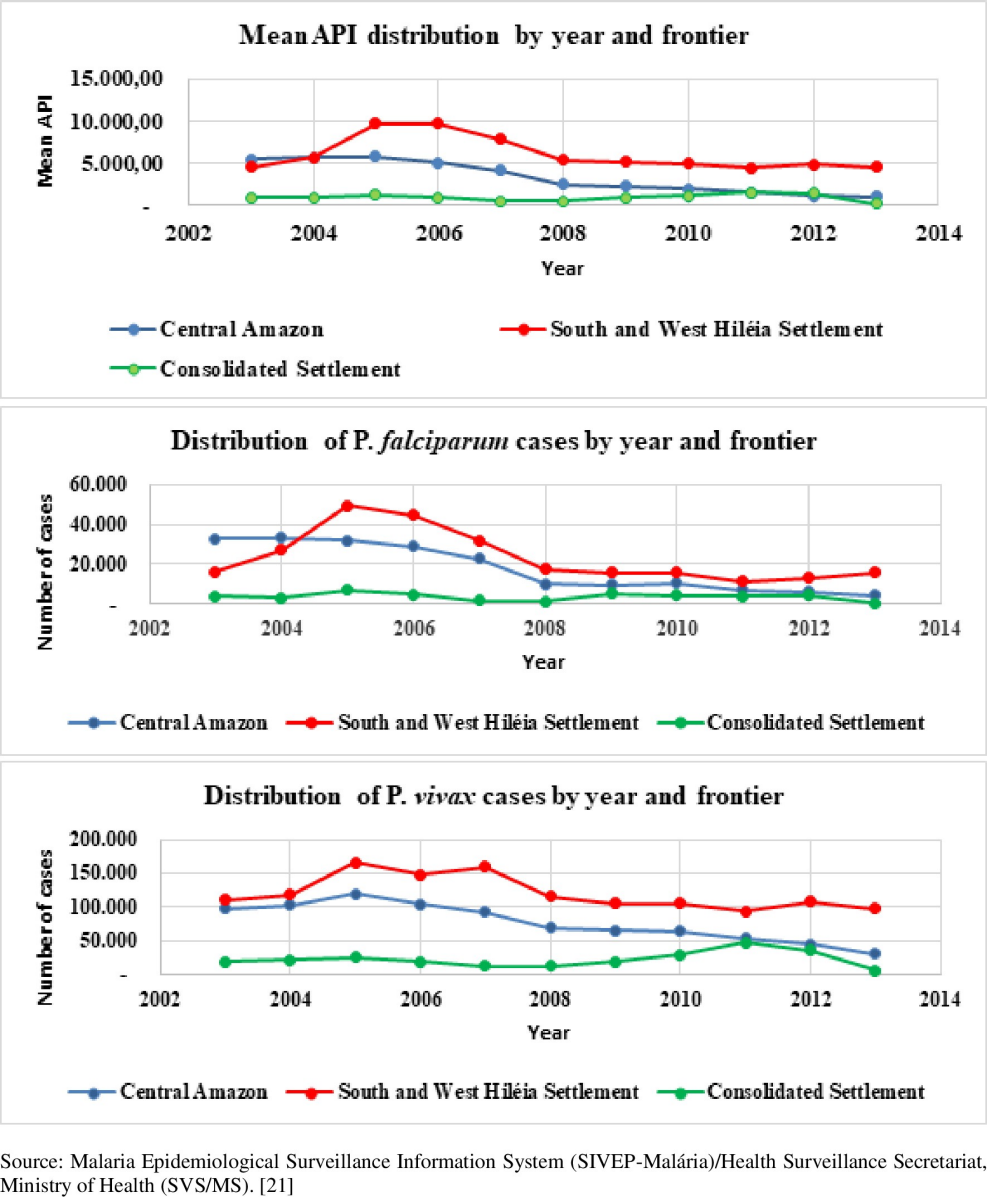
Mean API values and number of *P*. *falciparum* and *P*. *vivax* cases by year of diagnosis and regionalization. Brazilian Amazon, 2003–2013.

Fig 5 shows the temporal distribution of mean API values by type of regionalization. The south and west Hiléia settlement zone had the highest mean API for the entire period, with the highest concentration between 2004 and 2008 and a sharp decrease in 2007. This zone had the highest concentration of *P*. *falciparum* and *P*. *vivax* cases throughout the target period. However, *P*. *falciparum* cases showed the greatest proportional drop in 2008.

**Fig 6:**
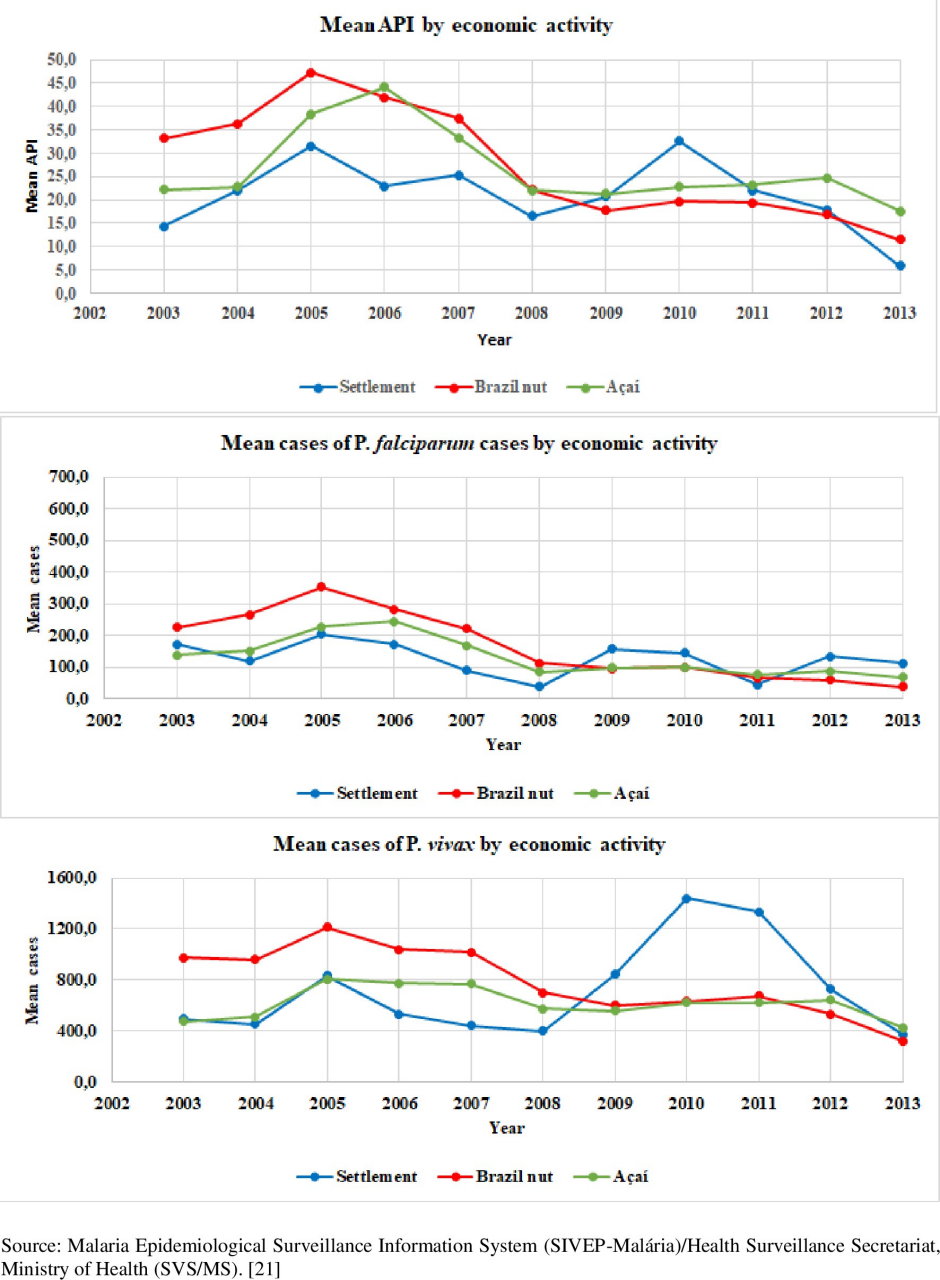
Mean API values of *P*. *falciparum*, and *P*. *vivax* by year of diagnosis and economic activity. Brazilian Amazon, 2003, 2005, 2010, and 2013.

Fig 6 shows the incidence distribution (total API, *P*. *falciparum*, and *P*. *vivax*) according to economic activity. The analysis of API by economic activity showed that in areas with Brazil nut production, there was a decrease in mean cases in 2005. For açaí extraction areas, the decrease started in 2006. Cases associated with agricultural settlements showed a wider variation, with peaks in 2005, 2007, 2009, and 2010. There was a smaller proportional variation in *P*. *falciparum* incidence according to economic activity. However, with regard to *P*. *vivax* incidence by economic activity, cases increased starting in 2008, with significant peaks in 2010 and 2011 in agricultural settlements, effectively, this represents the importance of the intermediate levels of SDH.

As shown in Figs 4 and 5, the malaria API patterns across the years and subregions was similar for total malaria and *Plasmodium* species. Similar results were observed in the association between malaria API and occupation, except for *P*. *vivax* from 2009 to 2011 (Fig 6).

Table 3 shows the results of the zero-inflated binomial regression models for the dependent variable *P*. *falciparum* cases and potential risk factors. In 2003, the presence of açaí production in a municipality was associated with a 1.98 times greater risk (95% CI: 1.40–2.84) of *P*. *falciparum* infection than municipalities without açaí production. Brazil nut production was associated with an increased risk of 2.49 times (95% CI: 1.69–3.67). In 2005, an epidemic year, açaí production was associated with a 1.86 times (95% CI: 1.22–2.85) greater risk of *P*. *falciparum*, and Brazil nut production was associated with a 2.25 times (95% CI: 1.43–3.55) greater risk. In 2010, there was an increased risk of *P*. *falciparum* with the presence of Brazil nut production [3.67 (95% CI: 2.34–5.75)] and vegetal product extraction[1.14 (95% CI: 1.07–1.21)], while road and dam construction was a protective factor, with a 0.74 times (95% CI: 0.67–0.82) lesser risk. In 2013, once again, açaí production served as a risk factor for increased cases of *P*. *falciparum*. Açaí production was associated with a 5.45 times (95% CI: 3.44–8.64) greater risk in municipalities with *Plasmodium* cases, compared to a 1.59 times greater risk for vegetal product extraction (95% CI: 1.24–2.05) and a 1.72 times greater risk for travelers (95% CI: 1.22–2.43).

**Table 3:** Risk factors of malaria associated with occupation for *P*. *falciparum* by years.

Variables	2003	2005	2010	2013
	RR (95% CI)	RR (95% CI)	RR (95% CI)	RR (95% CI)
**Distal Level**				
**Mega-projects** Roads and dams	-	-	0.74 (0.67–0.82)	-
Industrial mining	-	-	-	-
**Economic**	-	-	-	-
Per capita GDP	- -	-	-	-
**Regionalization**	-	-	-	-
Central Amazon	3.52 (2.39–5.19)	2.16 (1.41–3.29)	1.7 (1.08–2.69)	2.69 (1.58–4.56)
South and west Hiléia settlement zone	10.53 (6.40–17.35)	17.29 (9.95–30.04)	8.15 (4.50–14.75)	17.51 (9.23–33.21)
Consolidated settlement zone	1	1	1	1
**Intermediate Level**				
**Production**				
Açaí	1.98 (1.40–2.84)	1.86 (1.22–2.85)	-	5.45 (3.44–8.64)
Agricultural settlements	-	-	-	-
Brazil nuts	2.49 (1.69–3.67)	2.25 (1.43–3.55)	3.67 (2.34–5.75)	-
**Proximal Level** **Types of Occupation**	-	-	-	-
Tourism	-	-	-	-
Agriculture	-	1.02 (1.01–1.03)	1.04 (1.02–1.05)	1.05 (1.03–1.08)
Illegal mining	0.98 (0.98–0.99)	-	-	-
Vegetal product extraction	1.01 (1.01–1.03)	1.03 (1.01–1.04)	1.14 (1.07–1.21)	1.59 (1.24–2.05)
Travelers	-	0.97 (0.97–0.98)	-	1.72 (1.22–2.43)
**Gender**	-	-	-	-
Male	1.02 (1.01–1.02)	-	1.01 (1.01–1.02)	-
Female	0.98 (0.97–0.99)	-	-	-
Municipalities with malaria	369	349	263	161
Total cases	53,057	87,931	30,293	20,882
Total municipalities	771			

Note: Number of malaria cases was adjusted by zero-inflated negative binominal regression

Source: Malaria Epidemiological Surveillance Information System (SIVEP-Malaria)/Health Surveillance Department, Ministry of Health (SVS/MS). [23]

Table 4 shows the results of the zero-inflated binomial regression models for the dependent variable *P*. *vivax* cases and potential risk factors. In 2003, the only occupations associated with *P*. *vivax* cases were açaí and Brazil nut production, with a 1.01 times (95% CI: 1.01–1.02) greater risk; mega-projects sucha as industrial mining, with a 1.23 times (95% CI: 1.12–1.35) greater risk; and road and dam construction, with a 1.59 times (95% CI: 1.34–1.88) greater risk. Travelers in the area, a protective factor in 2003, became a risk factor with a 1.11 times (95% CI: 1.07–1.15) greater risk in 2010. In 2005, açaí production, with a 3.14 times (95% CI: 2.14–4.61) greater risk; agricultural settlements, with a 1.81 times (95% CI: 1.20–2.73) greater risk; and Brazil nut production, with a 1.55 times (95% CI: 1.01–2.38) greater risk, were positively associated with *P*. *vivax*, as were vegetal product extraction, with a 1.02 times (95% CI: 1.01–1.03) greater risk; industrial mining, with a 1.27 times (95% CI: 1.14–1.40) greater risk; and road and dam construction, with a 1.42 times (95% CI: 1.23–1.62) greater risk. In 2010, açaí production was associated with a 5.46 times (95% CI: 3.74–7.96) greater risk of *Plasmodium* transmission. A similar pattern was true for Brazil nut production, with a 3.85 times (95% CI: 2.55–5.83) greater risk; and agricultural settlements, with a 4.46 times (95% CI: 2.08–9.54) greater risk. In 2013, açaí production with 4.76 (95% CI: 3.23–7.01), Brazil nuts production with 2.05 (95% CI: 1.37–3.07), and agricultural settlements with 3.73 (95% CI: 1.44–9.62) were positively associated with *Plasmodium* incidence.

**Table 4:** Risk factors of malaria associated with occupation for *P*. *vivax* by years.

Variables	2003	2005	2010	2013
	RR (95% CI)	RR (95% CI)	RR (95% CI)	RR (95% CI)
**Distal Level**				
**Mega-projects**				
Roads and dams	1.59 (1.34–1.88)	1.42 (1.23–1.62)	-	0.85 (0.81–0.89)
Industrial mining	1.23 (1.12–1.35)	1.27 (1.14–1.40)	-	-
**Economic**				
Per capita GDP	-	-	-	-
**Regionalization**	-	-	-	-
Central Amazon	2.2 (1.57–3.07)	2.97 (2.01–4.42)	3.35 (2.31–4.86)	2.3 (1.54–3.43)
South and west Hiléia settlement zone	8.76 (5.59–13.83)	12.14 (7.35–20.05)	10.19 (6.03–17.23)	11.04 (6.48–18.77)
Consolidated settlement zone	1	1	1	1
**Intermediate Level**				
**Production**				
Açaí	-	3.14 (2.14–4.61)	5.46 (3.74–7.96)	4.76 (3.23–7.01)
Agricultural settlements	-	1.81 (1.20–2.73)	4.46 (2.08–9.54)	3.73 (1.44–9.62)
Brazil nuts	-	1.55 (1.01–2.38)	3.85 (2.55–5.83)	2.05 (1.37–3.07)
**Proximal Level**				
**Types of Occupation**				
Tourism	-	-	-	-
Agriculture	-	-	-	-
Illegal mining	-	-	-	-
Vegetal production Extraction	1.01 (1.01–1.02)	1.02 (1.01–1.03)	-	1.09 (1.01–1.18)
Travelers	0.98 (0.98–0.99)	-	1.11 (1.07–1.15)	-
**Gender**	-	-	-	-
Male	-	-	-	-
Female	-	-	-	-
Municipalities with malaria	542	506	435	346
Total cases	226,478	311,317	198,587	134,032
Total municipalities	771			

Note: Number of malaria cases was adjusted by zero-inflated negative binominal regression

Source: Malaria Epidemiological Surveillance Information System (SIVEP-Malaria)/ Health Surveillance Department. Ministry of Health (SVS/MS). [23]

Table 5 shows the results of zero-inflated binomial regression models for the dependent variable “all malaria cases” and potential risk factors. Regarding economic production, for all four years, the associations were similar to those for *P*. *vivax*. In 2003, the increased likelihood of malaria transmission was as follows: municipalities with açaí production had a 3.63 times (95% CI: 2.61–5.05) greater risk, and municipalities with Brazil nut production has a 2.63 times (95% CI: 1.81–3.83) greater risk. For occupations, there was a positive association with industrial mining, 1.20 (95% CI: 1.12–1.27), and road and dam construction, 1.54 (95% CI: 1.36–1.75). In 2005, açaí production and Brazil nut production were associated with increases in malaria incidence on the order of 4.73 (95% CI: 3.19–7.01) and 2.02 (95% CI: 1.34–3.05), respectively. The work most significantly associated with malaria was road and dam construction (1.21; 95% CI: 1.08–1.36). In 2010, malaria was associated with açaí production, with a 4.77 times (95% CI: 3.27–6.97) greater risk; agricultural settlements, with a 4.20 times (95% CI: 1.97–8.96) greater risk; and Brazil nut production, with a 4.27 times (95% CI: 2.83–6.43) greater risk. In 2013, Brazil nut production remained positively associated with malaria, with a 3.00 times (95% CI: 1.95–4.60) greater risk, while road and dam construction (0.84, 95% CI: 0.81–0.89) served as a protective factor.

**Table 5:** Risk factors of malaria associated with occupation for all *Plasmodium* species by years.

Variables	2003	2005	2010	2013
	RR (95% CI)	RR (95% CI)	RR (95% CI)	RR (95% CI)
**Distal Level**				
**Mega-projects**				
Roads and dams	1.54 (1.36–1.75)	1.21 (1.08–1.36)	-	0.84 (0.81–0.89)
Industrial mining	1.20 (1.12–1.27)	-	-	-
**Economic**	-	-	-	-
Per capita GDP	-	-	-	-
**Regionalization**	-	-	-	-
Central Amazon	2.44 (1.73–3.43)	2.37 (1.58–3.55)	2.88 (1.99–4.18)	1.40 (0.93–2.08)
South and west Hiléia settlement zone	5.50 (3.55–8.52)	9.22 (5.62–15.14)	9.32 (5.51–15.77)	7.25 (4.15–12.66)
Consolidated settlement zone	1	1	1	1
**Intermediate Level**				
**Production**				
Açaí		4.73 (3.19–7.01)	4.77 (3.27–6.97)	-
Agricultural settlements	-	-	4.20 (1.97–8.96)	-
Brazil nuts	2.63 (1.81–3.83)	2.02 (1.34–3.05)	4.27 (2.83–6.43)	3.00 (1.95–4.60)
**Proximal Level**				
**Types of Occupation**				
Tourism	-	1.10 (1.06–1.13)	-	-
Agriculture	-	-	-	-
Illegal mining	-	-	-	-
Vegetal production Extraction	-	-	1.09 (1.06–1.12)	-
Travelers	-	-	-	-
**Gender**	-	-	-	-
Male	-	-	-	-
Female	-	-	-	-
Municipalities with malaria	560	524	449	390
Total cases	292,590	419,095	236,243	164,484
Total municipalities	771			

Note: Number of malaria cases was adjusted by zero-inflated negative binominal regression.

Source: Malaria Epidemiological Surveillance Information System (SIVEP-Malaria)/Health Surveillance Department, Ministry of Health (SVS/MS). [23]

## Discussion

Malaria incidence in the Brazilian Amazon has dropped significantly in the last decade, with the number of reported cases decreasing by half (Siqueira et al. 2016). However, malaria control measures have not been as successful in the south and west Hiléia settlement zone. Moreover, “frontier malaria” has continued to expand in municipalities located in the expansion region of the agricultural frontier [22] due to a combination of biological, environmental, and socioeconomic factors favorable to transmission [35] [36] [26]. Historically, malaria has been controlled or eliminated in various places, only to reemerge with even greater force due to several factors [37]. This illustrates the vulnerability of this process and highlights the importance of understanding the disease transmission dynamics over time in different settings to maintain the gains achieved by malaria control and elimination programs [22] [38].

According to the findings, most of the malaria-positive municipalities were concentrated in the south and west Hiléia settlement zone, where a marked API increase occurred between 2004 and 2008. This zone experienced a marked decrease compared to the other regions in 2009 and a higher concentration of *P*. *falciparum* and *P*. *vivax* cases throughout the period, with the most significant risk for *P*. *vivax*. Interestingly, contrary to previous studies [22] [3]and partly as a consequence of the process of occupation of the economic frontier, most of the areas where malaria occurred (formerly areas with forest fringes, industrial mining, illegal mining, agriculture, road construction, deforestation, and açaí and Brazil nut extraction) were concentrated along the Amazon River and in the extractive reserves [39] [22] [40]. This study aimed to integrate evidence-based disease surveillance and control by ranges or zones of territorial occupation of the economic frontier according to economic activities.

The National Malaria Control Program (PNCM), which establishes disease control policy, increased its activities during the study period. Programs to reduce malaria morbidity expanded in 2015 compared to the relatively few programs in 2000.

The study also showed a clear lack of synchrony between the prevailing malaria control programs and the occupation model of the pioneer fronts, as there are still specific geographic locations favoring disease transmission. Frontier malaria usually occurs in locations with weak institutions, minimal community cohesiveness, political marginalization of settlers, and high rates of internal and external migration, with sufficient human mobility to ensure the spread of *Plasmodium* spp. [22]. Malaria in these areas is closely associated with deforestation and agriculture and with the unplanned development of new agricultural settlements and is facilitated by the failures of former settlements and by settlers’ attempts to avoid new episodes of the disease [40]. This dynamic setting combined with these conditions severely limits organizational attempts to minimize the malaria risk in tropical ecosystems and serves to promote disease transmission.

### Malaria risk according to economic activity

The extractive harvesting of açaí and Brazil nuts is associated with an increased risk of malaria transmission, especially *P*. *falciparum*. According to our results, farmers or agricultural workers and travelers showed a nearly two-fold increased risk of *P*. *falciparum* infection. Meanwhile, there was a gradual decrease in malaria incidence associated with industrial mining, illegal mining, and road and dam construction, and these activities appeared to act as a protective factor against *Plasmodium* infection in the respective municipalities. Several international studies have shown an increased risk of malaria transmission in rural areas where people are involved in extractive activities [22] [25] [3] [14] [38] [41] [40] [42] [43] [44] [24]. In the Brazilian Amazon, a survey in 2000–2005 concluded that municipalities where 0.7% of the remaining forests were selectively exploited had a higher risk of malaria (1.72, 95% CI: 1.18–2.51) compared to areas with the highest selective logging rates, which had a lower risk (0.39, 95% CI: 0.23–0.67) (Hahn, Gangnon, Barcellos, Asner, & Patz, 2014). Several studies have also documented malaria transmission in agricultural settlements in the Amazon, emphasizing the importance of differentiated control measures according to the stage of the settlement project (recent or past implementation) and the levels of transmission in each location [22] [25] [3] [14] [38] [41] [40] [42] [43] [44] [24]. More recently, another study found evidence that proximity to forest fringes increases the malaria incidence, especially in locations close to environmental protection areas (EPA) where plant extractive activities usually occur [45].

In the south and west Hiléia settlement zone, the risk and exposure to malaria in açaí extractive or cultivated production areas varies by region. For example, in the Amazonian estuary, the risk increased during the peak harvest season in July and August. From November to May, the possible areas for malaria transmission are those with cultivated açaí in the state of Amazonas (Codajás, Itacoatiara, Careiro, Urucará, Parintins, and Maués) and with extractive açaí activities (Tefé, Coari, Codajás, and Manaquir). In Amapá, the high-risk areas are concentrated in Mazagão, Serra do Navio, Pedra Branca, Santana, and Macapá from August to December. Risk occurs year round in the municipalities of Cruzeiro do Sul, Mâncio Lima, Marechal Thaumaturgo, Porto do Acre, and Rodrigues Alves in the state of Acre, and in the state of Rondônia, the risk is associated with cultivated açaí from January to June in Porto Velho [46].

Açaí and Brazil nut extraction and cultivation are mostly concentrated in rural floodplains [47] [48] that favor the development and permanence of immature anophelines, mainly *Anopheles darlingi*, the principal malaria vector in the Amazon [49]. Water samples collected from forest fringes were more likely to be positive for *An*. *darling* larvae [49].

In the south and west Hiléia settlement and Central Amazon zones, the highest malaria incidence occurred in areas with Brazil nut extraction, located mainly in the states of Acre, Amazonas, and Pará, where the annual harvests occur. In Roraima, the harvest season runs from March to September since meteorological conditions differ from the other Amazonian states. The states of Maranhão, Mato Grosso, and Tocantins also have some areas with Brazil nut extraction. Health services must focus on detection in December or January, except in Roraima where detection is from March to September, due to the environmental conditions and set goals for the containment of epidemics in these extractive production zones [46].

This regional analysis of the Brazilian Amazon showed differences in the spatial distribution of malaria, clearly determined by the methods of territory occupation spatially and over time. *P*. *falciparum* and *P*. *vivax* cases associated with potential risk factors over time were used to assess malaria trends associated with occupation of the Amazonian frontier. Considering the three main zones of occupation in the Amazon, there was only a marked increase in malaria risk in the south and west Hiléia settlement zone between 2003 and 2013. In 2005, this subregion showed a 12.14 times (95% CI: 7.35–20.05) greater risk for *P*. *vivax* infection. In 2013, the increased risk was 17.51 times (95% CI: 9.23–33.21) greater in municipalities with *P*. *falciparum* cases. This overview of regions with increased risk of malaria highlights the importance of integrating evidence-based surveillance and disease control by ranges or zones of territorial occupation along the economic frontier.

This study emphasizes the importance of malaria risk according to territorial occupation zones. Each zone, with its individual economic specialization, has areas and individuals who can be affected differently by the dynamics of the health-disease process. This study highlights common characteristics of the localities that help explain how the disease behaves depending on the prevailing environmental conditions. For example, in the south and west Hiléia settlement zone (the new mobile frontier for the exploitation of natural resources), malaria’s spatial dispersion displays marked heterogeneity. In this subregion, malaria is strongly associated with deforestation and subsistence agriculture due to the unplanned development of new agricultural settlements [45].

One hypothesis suggests that the decreasing malaria incidence is associated with new forms of occupation of the economic frontier, with export-oriented market mechanisms. This is paralleled by the frontier workers’ mobility due to the lack of governmental policies for organized territorial settlement. This mobility increases the frontier workers’ vulnerability, with greater risk of exposure to infection. These findings corroborate previous studies emphasizing the role of mobility and area closure (postfrontier rationale) in the spatial dispersion of malaria in the postfrontier stage, probably due to a combination of the following: (i) expanded mechanized soybean monoculture, extensive agriculture, energy infrastructure projects (e.g., the Madeira and Belo Monte hydroelectric dams), and industrial mining [46]; (ii) the establishment of protected areas and reduced deforestation regions [50] [42]; (iii) the introduction of cattle grazing as part of established soybean and corn crop rotations [50], leading to the further concentration of land ownership and thus clearing fields and transferring infection risks to periurban areas [22]; and (iv) the development of local surveillance and health care systems [43].

### Mobility, spatial spread, and malaria

An important development in the Brazilian Amazon was reduced interregional migration, suggesting that migration in the region was driven by specific projects associated with recent Federal government policies such as “*Brasil em Ação”* (Brazil in Action) and the Growth Acceleration Program (PAC) [19] [51]. In other words, migration in the region was determined by specific projects that potentially attracted Brazilians to the states of Amazonas, Acre, Amapá, and southwest of Pará, all of which experienced growing populations [52]. There has also been significant population growth in urban areas, especially regional urban hubs and medium-sized cities [53].

The south and west Hiléia settlement and Central Amazon zones showed a malaria risk in all the years studied. However, there was a decrease in cases due to reduced economic activity in the migratory frontiers in recent years, meaning the Brazilian Amazon had a lower rate of migration to rural areas. Highly extensive agricultural activities (livestock, soybeans, cotton) have led to a reduced labor force and reduced migration to rural areas [54]. Meanwhile, the region showed stable urbanization during the same period, with towns and cities becoming the last option for migrants (a situation further aggravated by poor urban infrastructure, work and employment conditions in the region’s towns and cities) [53].

Public services were effective in the consolidated settlement zone, which had large cities and a dense road network. This zone did not contribute to malaria spread in the region due to the reduced natural habitats for anopheline mosquito reproduction.

The current study corroborates several previous studies on malaria spread and mobility [55] [56] [57], showing that human migration to endemic malaria areas increases the risk of infection by thirty-fold. There are three stages in the spread of frontier malaria [8]: the first stage is marked by a rapid increase in malaria incidence in the early years of agricultural settlement projects. The second stage shows a progressive decline in malaria rates after three years due to improved environmental and socioeconomic conditions in agricultural settlements. Finally, the third stage is stability, with low malaria incidence as a result of urbanization, increasing sense of community, improved socioeconomic conditions, and a reduction in environmental changes. In 2003, the contiguous distribution of malaria occurred in the Central Amazon and south and west Hiléia settlement zones. This contiguous distribution and subsequent reduction were evident in the 2005 epidemic in the south and west Hiléia settlement zone; in 2013, malaria distribution was concentrated in the south and west Hiléia settlement zone, specifically in the states of Amapá, Amazonas, and Roraima [35].

In the current study, municipalities with important agricultural colonization and environmental protection areas for açaí and Brazil nut extraction were associated with the spread of malaria. In these areas with regular and intense human circulation, the proportion of recent immigrants is assumed to be large, as is the proportion of young men in the population. These municipalities are characterized by immigration and substandard living conditions; therefore, these areas are socially vulnerable to malaria and maintain endemicity with high incidence rates [22] [14] [58] [44]. Forest conservation in environmental protection areas can increase the disease prevalence in the vector species [24] [59] [60], while the risk for transmission is reduced in deforested areas of the Brazilian Amazon, which is generally covered by shrubs and secondary vegetation, where forests tend to be replaced by pastures and soybean crops [61].

The observations of the economic activities and social mobility showed that malaria risk was highest in individuals working in vegetal product extraction (açaí and Brazil nuts) and agricultural settlements, the latter specifically in the south and west Hiléia settlement zone. The highest malaria risk for vegetal extraction activities was in young men, corroborating several other studies and reflecting social determination and gender asymmetry in malaria incidence. The literature shows that work near forest areas is mostly done by men and that the mean number of malaria cases per month and per person tends to be higher in men than in women [50].

Importantly, malaria risk can be associated with situations of social stratification caused by differential access to land (i.e., workers’ activity on the economic frontier according to land tenure and transformations in soil use observed over time) and the social mobility of individuals or families. In the Brazilian Amazon, trends in human mobility are not strictly linear. Many people are in a cyclical transition between different stages of social stratification and displacement on the economic frontier, and they can often be categorized differently at different times; for example, a poor migrant with the social status of an illegal miner can become a settler or landless squatter, or a settler can transform from small farmer to medium-sized or large cattle rancher [62].

Circular migration in the Amazon is highly common. It occurs when the land, labor, services, markets and preexisting social structures are marked by the concentration of land and natural resources, fueling the marginalization of people and social inaccessibility, facilitating poor living conditions and a higher likelihood of displacement, social mobility, and the development of adverse health conditions for malaria [54] [63] [64].

### Malaria control on the Amazonian frontier

The implementation of the National Malaria Control Program (PNCM) in 2003 led to fluctuations in transmission of the disease, and 2003 and 2005 were marked by periods of major epidemics followed by periods of reduced disease spread. Brazil achieved the Millennium Goal in 2015 with a reduction in malaria cases of more than 75% compared to the number of cases in 2000. In the last 15 years, the country has reported an average of 320,000 annual cases, which is 50% of all malaria cases in the Americas [1].

The PNCM intensified its malaria control activities with an important increase in the network of microscopy laboratories, a free supply of long-lasting insecticide-treated mosquito nets (ITNs), indoor spraying focused in areas with increased risk, and improved community awareness and education [65].

The *P*. *vivax/P*. *falciparum* ratio in Brazil increased from 3.8:1 in 2003 to 4.9:1 in 2013 and 7.8:1 in 2016. The ratio was approximately 1:1 until 1989. The introduction of combination therapies with artemisinin derivatives (ACTs) for the treatment of *P*. *falciparum* in 2007 was undoubtedly instrumental in reducing malaria cases in the country [66]. The expanded diagnostic network and inclusion of rapid diagnostic tests (RDTs) contributed to this decline by interrupting the *P*. *falciparum* transmission chain, since *P*. *falciparum* takes 7 to 10 days after the onset of symptoms to produce infectious gametocytes [39].

In 2003, the Brazilian Ministry of Health implemented an information system for the epidemiological surveillance of malaria (SIVEP-Malaria), which allows disease case monitoring and real-time updating by municipal health systems, although this does not always occur. Although the system is simple, it is not always used by municipal managers to assess the epidemiological situation and issue prompt alerts at the onset of epidemics. A study in 2010 using SIVEP data showed that epidemics occurred in 41.9% of the municipalities in the endemic area, lasted 1–4 months in 58.3% of the municipalities, 5–8 months in 24.3% of the municipalities, and 9–12 months in 17.4% of the municipalities [39]. For control and elimination of the disease, adequate use of this information system can be key for assessing the epidemiological situation, detecting the early onset of epidemics and observing their main determinants to contain outbreaks and make appropriate response decisions.

Regardless of the frontier workers’ mobility, moving from deforested areas to environmental protection areas exposes workers to different risk situations and thus requires close monitoring by malaria control policymakers.

### Study limitations

The study had three important limitations. First, it drew mainly on secondary data, with the potential for inaccurate information and omission of relevant variables. More than one positive test may have been performed and reported for the same malaria case (overreporting), and some cases may not have been reported due to an incorrect diagnosis. Second, the annual parasite incidence (API) was adopted as an indicator of malaria transmission risk and was used to classify areas by an estimated disease incidence rate. The API consisted of counting positive tests rather than actual cases of the disease. Third, since the API spatial interpolation only provided an estimate of a value based on other known values, the distribution of malaria cases and low data density in some municipalities may have impacted the quality of the results. Additionally, the resolution of the interpolation grids must be consistent with the resolution of the sample grids. We emphasize that these three limitations are typical of studies conducted on regional geographic scales, demonstrating the difficulty in determining inherent relations between local dynamics and broader regional scales, the results of which may or may not be generalizable to a broader spatial context and may overlook many of the local complexities of malaria transmission.

## Conclusion

The study aimed to elucidate the associations between types of economic occupation, social mobility, and regionalization of territorial occupation and the incidence of malaria. Analyzing the dynamics of infection by type of economic activity, *Plasmodium* species, and modeling strategies at the regional scale, we aimed to observe the spatial-temporal variations in malaria rates to identify specific patterns in the transmission of the disease in different areas of territorial occupation.

Describing malaria in the postfrontier stage of the Brazilian Amazon assumes that the disease displays different temporal and spatial dissemination patterns, which are driven by the replacement of high incidence rates caused by occupation and ecological transformations due to local deforestation combined with human migration. These conditions are altered by processes of urbanization, which are interventions (especially with the introduction of impermeable surfaces and drains) that create environments that are inhospitable to *Anopheles darlingi* larvae and are increasingly removed from the forest fringes, thereby substantially reducing human exposure to the disease [67]. Currently, malaria is spatially concentrated in hotspots along Amazon River channel and in the south and west Hiléia settlement zone, where environmental protection areas and açaí and Brazil nut extraction are concentrated. Thus, this study characterized spatial patterns of disease transmission according to the economic activities and regionalization of geographic spaces, confirming that infection is associated with productive activity and labor flow linked to extractive activities and agricultural settlements, which can be a useful tool in malaria surveillance for mapping risk areas.

We identified four distal socioenvironmental determinants of malaria (Distal Level) that act as economic drivers interrelated with the risk of malaria in the Brazilian Amazon: roads, and dam construction, industrial mining, Central Amazon and south and west Hiléia settlement zone. However, the data suggest that the risk is persistently higher in the areas of the south and west Hiléia settlement zone in each year and for each of the Plasmodium species and somewhat lower in the Central Amazon. Although, large projects as roads and dams construction and industrial mining are also associated with the risk of malaria, strength of association is lesser and it was not observed in all years and for all species studied. In fact, a couple of years it appears as a protective factor. These factors increase the likelihood of malaria transmission, considering that the dynamics of the malaria health-disease process can be affected differently each zone according to territorial occupation, with its individual economic specialization. The associations of intermediate level which corresponds to malaria exposure proxies can be reduced to three distinct but interrelated social determinants: açaí and Brazil nuts commercial production and agricultural settlements appears persistently associated with the risk of becoming ill in practically every year and for all Plasmodium. This risk is not so high for agricultural settlements that was associated with of the disease by P. vivax but not for P. falciparum. In the three cases, that kind of exposure actually increased the access of workers to regions at risk of malaria, displacement to receptive areas that proximity to forest fringes, such as substandard living conditions, contact increase with Anopheles vectors and high immigration of infected populations. Finally, the proximal level corresponds to individual characteristics that influence of malaria risks. In the current study, it was found as a determinant of proximal risk, a forestry, as occupation which was seen in P. vivax and P. falciparum in practically all studied years, despite being weakly associated with malaria. Travelers and tourism, are known to be at greater risk of malaria, however, it should be studied in more depth as well as male individuals which has been associated with the risk to get sick for P. falciparum, but weakly. We postulate that the risk to individuals working is greater because they disproportionately live situations of social stratification caused by differential access to land and the social mobility of individuals or families. If young men in the population move in endemics areas, with high incidence rates and adverse social conditions, they might experience other social determinants, reflecting social determination and gender asymmetry in malaria incidence.

## Supporting information

S1 Table Annual Parasite Index (API) and *Plasmodium* species according to year and regionalization. (XLSX)
